# Purple Corn Extract Prevents Doxo-Induced Cardiotoxicity by Counteracting AMPK Activation and p53 Acetylation in HL-1 and Primary Cardiomyocytes

**DOI:** 10.1155/omcl/7786043

**Published:** 2025-09-18

**Authors:** Francesca Cappellini, Debora Zorzan, Federica Tomay, Marta Toccaceli, Alessandra Marinelli, Marina Mancini, Annalisa Bucchi, Chiara Tonelli, Katia Petroni

**Affiliations:** Dipartimento di Bioscienze, Università degli Studi di Milano, Milan, Italy

**Keywords:** AMPK, anthocyanins, cardioprotection, doxorubicin, p53 acetylation

## Abstract

Doxorubicin (Doxo) is an anthracycline widely used as a chemotherapeutic agent for many solid and hematological cancers. Its clinical use is limited due to a cumulative dose-dependent and irreversible cardiotoxicity that can cause progressive cardiomyopathy and congestive heart failure. A cardioprotective therapy that can decrease heart damage without reducing the anticancer efficacy during Doxo therapy is of utmost importance. Anthocyanins (ACNs) are renowned cardioprotective agents thanks to their antioxidant and anti-inflammatory properties. An ACN-rich diet from purple corn, which mainly contains cyanidin 3-glucoside (C3G) and its acetylated derivatives, has been previously shown to be effective in reducing Doxo-induced cardiotoxicity in mice. Aiming at unveiling the molecular mechanisms involved in ACN protection, we considered the fibroblast growth factor 21/AMP-activated protein kinase/SIRTUIN1 (FGF21/AMPK/SIRT1)/p53 pathway in murine HL-1 cardiomyocytes treated with Doxo in the presence or absence of purple corn extract (RED). Our work shows that Doxo-induced AMPK activation is restored to control levels by the RED extract. p53 acetylation was increased by the RED extract and upon Sirt1 silencing, indicating that p53 acetylation is SIRT1-dependent and suggesting that the RED extract may affect SIRT1 activity through AMPK. Notably, increased p53 acetylation led to decreased levels of cleaved-caspase 3 and Puma and p21 transcript levels, indicating a reduced level of apoptosis. The RED-induced cardioprotection and p53 acetylation were confirmed in mouse primary cardiomyocytes. In conclusion, the RED extract may prevent cardiomyocytes apoptosis through the modulation of AMPK and acetylation of p53.

## 1. Introduction

Doxorubicin (Doxo) is one of the most used anticancer drugs, but one of its major side effects is cardiotoxicity, which can occur in an acute form or as a progressive heart failure with irreversible cardiac dysfunction [1]. Multiple mechanisms have been proposed for Doxo-induced cardiotoxicity, with the common endpoint being cardiomyocyte death through autophagy or apoptosis [2–4].

Sirtuins are a class of deacetylases that have been proposed to play a role in Doxo-induced progressive heart failure. In particular, SIRTUIN1 (SIRT1) plays a major role in cardiac development, since *Sirt1*^−/−^ mice rarely survive postnatally. Patients with advanced heart failure show a reduction in *Sirt1* expression. Several studies on cardiac cell lines have reported that Doxo decreases SIRT1 protein levels and/or activity, while increased SIRT1 activity reduces Doxo-induced cardiac dysfunction [5].

Recently, fibroblast growth factor 21 (FGF21), a target of SIRT1, has been demonstrated to be a key player in cardiac remodeling. Although FGF21 is considered a hepatic hormone, it is also produced in the heart, where it exerts a protective role. In cardiomyocytes, FGF21 also acts through an autocrine mechanism: secreted cardiac FGF21 activates SIRT1, which in turn promotes FGF21 synthesis [6]. A recent study reported that FGF21 restores AMP-activated protein kinase (AMPK) activity in cardiomyocytes through the activation of the AMPK/SIRT1/p53 pathway [7]. p53 is another SIRT1 target involved in Doxo-induced cardiotoxicity. SIRT1-mediated p53 deacetylation inactivates p53 transcriptional activity and represses p53-mediated apoptosis [8]. AMPK promotes dissociation of SIRT1 from its inhibitor DBC1 by direct phosphorylation of SIRT1, leading to SIRT1 activation and p53 deacetylation [9].

Anthocyanins (ACNs) are polyphenols belonging to flavonoids and have been highlighted for their beneficial effects on a multitude of diseases. In particular, ACNs can prevent or reduce cardiovascular diseases and the risk of myocardial infarction in humans [10, 11]. ACNs have demonstrated a protective role in mice and rats against Doxo-induced cardiotoxicity without affecting its antitumoral activity in vitro [12–14]. The aim of this work was to uncover the molecular mechanism behind ACN-mediated cardioprotection against Doxo toxicity, focusing on the FGF21/AMPK/SIRT1/p53 axis.

## 2. Material and Methods

### 2.1. Purple Corn Extract

Purple corn extract (RED) was produced by SVEBA Srl (Como, Italy) from *B1 Pl1* purple corn cobs, as previously described [15, 16]. The RED extract was obtained through extraction with ethanol:H_2_O (30:70 *v*/*v*) at 55°C for 1 h, titration to a concentration of 4% ACNs, and spray-drying to a final concentration of 31.25 mg/g of ACNs. As previously described, the RED extract mainly contains cyanidin 3-glucoside (C3G) and its acylated derivatives (cyanidin 3-malonylglucoside and cyanidin 3-dimalonylglucoside), and to a lesser extent, pelargonidin 3-glucoside, peonidin 3-glucoside, and their acylated derivatives (pelargonidin 3-malonylglucoside and peonidin 3-malonylglucoside) [14, 15].

### 2.2. HL-1 Cell Culture and MTT Assay

HL-1 cardiomyocytes were cultured in Claycomb medium supplemented with 10% FBS, 100 µg/mL of penicillin/streptomycin, 0.1 mM norepinephrine, and 2 mM L-glutamine (Sigma). The flasks were precoated with gelatin/fibronectin (0.02% of gelatin; 5 μg/mL fibronectin, Sigma). Cells were maintained in a humidified incubator at 37°C containing 5% CO_2_. Cell viability was evaluated by MTT (3-(4,5-dimethylthiazol-2-yl)-2,5-diphenyltetrazolium bromide) assay, as previously described [14]. Cells were seeded in 96-well plates at a density of 40,000 cells/well for 24 h and treated with different concentrations of Doxo (0.25 μM, 0.5 μM, and 1 μM; Accord Healthcare) in the presence or absence of RED (125 μM) for 48 h. Cells were then incubated with 1% of MTT solution per volume for 2 h and solubilized in 0.1 N HCl, isopropanol, 10% Triton X-100. Optical density was measured using a microplate reader (Tecan Infinite F200PRO) at a wavelength of 570 nm.

### 2.3. Isolation of Murine Primary Cardiomyocytes

Adult ventricular cardiomyocytes were isolated from 2 to 3 month-old male WT mice (C57Bl/6J, Charles River), following the protocol described by Ackers-Johnson and Foo [17] with some modifications. All the procedures on animals were in accordance with Italian law (D. Lgs n 2014/26, implementation of 2010/63/UE) and approved by the Italian Ministry of Health (Project Number 839C7.N.AD7). All mice were maintained under pathogen-free conditions with 12 /12 h light/dark cycles. Mice were euthanized by cervical dislocation, and the heart was rapidly excised and put into Tyrode's solution (140 mM NaCl, 5.4 mM KCl, 1.8 mM CaCl_2_, 1 mM MgCl_2_, 0.1% glucose, HEPES NaOH 5 mM, and pH 7.4) supplemented with heparin 10 U/mL (Sigma H3393). Under a stereomicroscope, on ice and in Tyrode + heparin 10 U/mL, the heart was cleaned, exposing the aortic arch. The aorta was cannulated using an insulin needle, and Tyrode + heparin 10 U/mL was injected. The heart was then perfused according to the Langendorff model. An EGTA solution composed of basic Tyrode (130 mM NaCl, 5.4 mM KCl, 0.4 mM NaH_2_PO_4_, 0.5 mM MgCl_2_, 25 mM Hepes, 22 mM glucose, and pH 7.0) plus EGTA NaOH (0.23 mM) was perfused for 3 min. Then, the enzymatic solution composed of basic Tyrode, collagenase type 2 (220 U/mL, Worthington LS004176), and CaCl_2_ (0.1 mM) was perfused for 5 min and 30 s. The atria were then removed, and the ventricles were mechanically dissociated in the enzymatic solution. The heart fragments were transferred into a 15 mL tube with the perfusion buffer (130 mM NaCl, 5 mM KCl, 0.5 mM NaH_2_PO_4_, 10 mM HEPES, 10 mM glucose, 10 mM BDM, 10 mM Taurine, 1 mM MgCl_2_, and pH 7.8, sterile filtered) and mechanical dissociation was continued by pipetting up and down under a sterile hood. Cells were centrifuged at 300 rpm for 3 min, resuspended in perfusion buffer, and allowed to pellet for 20 min. Calcium readjustment was performed using three solutions with increasing calcium concentrations. Cells were resuspended in a solution containing 75% perfusion buffer and 25% culture medium (Medium 199—Gibco 11150-059—supplemented with 0.1% BSA, 1x ITS—Sigma I3146, 10 mM BDM—Sigma B0753, and 1x penicillin/streptomycin), and allowed to pellet for 20 min. The supernatant was removed, and the same procedure was repeated with a solution containing 50% perfusion buffer and 50% culture medium, followed by a solution containing 25% perfusion buffer and 75% culture medium. Cells were then resuspended in plating medium (Medium 199 supplemented with 5% FBS—Gibco, 10 mM BDM, and 1x penicillin/streptomycin) and plated on Matrigel (Corning) precoated dishes. After 1 h and 30 min, the medium was changed and the cells were maintained in culture medium. Cells were maintained in a humidified incubator at 37°C containing 5% CO_2_, and the culture medium was changed daily. Cell viability was calculated as a percentage of the number of living rod-shaped cells at 0 h of treatment divided by the number of living cells at 24 or 48 h of treatment.

### 2.4. Flow Cytometry

HL-1 cells were plated at a density of 50,000 cells/well in 96-well plates with 0.5 μM Doxo for 24 h and treated for 48 h with or without 125 μM RED. After treatment, HL-1 cells were trypsinized, four wells for each treatment were pooled and resuspended in PBS at a concentration of 1,000,000 cells/mL, and labeled with 5 μL Annexin V-AF488 (Molecular Probes) for 15 min. After the addition of 1 μL propidium iodide, samples were analyzed by flow cytometry. To determine *Δψm*, 1,000,000 HL-1 cells were suspended in medium and incubated with 50 nM tetramethylrhodamine ethyl ester (TMRE; Molecular Probes) for 20 min at 37°C. Cells were then washed in Hank's balanced salt solution, resuspended in FACS buffer (0.2% BSA in PBS), and kept on ice until analysis on a BD FACS CantoII with FACSDiva 6.1.1 software (Becton Dickinson, US). Two independent biological experiments were performed.

### 2.5. Mini-Gel Comet Assay

HL-1 cells were seeded in 24-well plates (30,000 cells/well) 24 h before exposure to 0.25 and 0.5 µM Doxo with or without 125 µM RED for 3 and 24 h in complete medium. Cells were washed twice with PBS and 50 µL of 0.05% trypsin/EDTA (T3924, Sigma) were added for 3 min at 37°C. Then, 50 µL of trypsin inhibitor (Soybean trypsin inhibitor, T6522, Sigma) and 150 µL of wash medium (Claycomb medium with 5% FBS e 1% penicillin/streptomycin) were added. A 30 μL of the cell suspension was mixed with 300 µL of 1% low-melting-point agarose (A9414, Sigma) at 37°C. A15-μL aliquots were dropped onto two different slides precoated with 0.5% agarose (A9539, Sigma). Each slide contained eight drops. Lysis, unwinding, electrophoresis, and staining steps were performed as previously described [18]. For each sample, at least 100 cells were scored using a fluorescence microscope (Zeiss Axiophot D1), and the results were expressed as damage index (with a value between 0 and 400), calculated by dividing the comets into four classes of increasing damage and using the formula: [(*n*×0) + (*n*×1) + (*n*×2) + (*n*×3) + (*n*×4)], where *n* is the number of comets within each class of damage.

### 2.6. Silencing

Silencing of HL-1 cells was performed using Lipofectamine RNAiMAX reagent (Thermo Fisher Scientific) following the manufacturer's instructions. HL-1 cells were seeded in a gelatin/fibronectin precoated 12-well plate at a density of 400,000 cells/well in 800 µL of complete medium. Cells were immediately silenced by adding 200 µL of a mixture of Lipofectamine RNAiMAX/siRNA solution in Opti-MEM medium (Thermo Fisher) containing 20 pmol of siRNA (QIAGEN) and 7.5 µl Lipofectamine RNAiMAX. After 24 h, cells were treated with 0.5 or 1 µM of Doxo with or without 125 µM RED for 48 h. The supernatant was centrifuged at 10,000 × *g* for 10 min and stored at −20°C. Total RNA and proteins were extracted as described below. For each gene silenced, two different siRNAs targeting two different RNA sequences were used (Supporting Information 1: Table S1).

### 2.7. RNA Extraction and Real-Time RT-PCR

Cells were lysed using QIAzol (QIAGEN) and total RNA was extracted using the Direct-zol RNA MiniPrep kit (Zymo Research), following the manufacturer's protocol. For each sample, 1 μg of total RNA was reverse transcribed using a patented mixture of oligodT and random primers with the iScript cDNA Synthesis kit (Bio-Rad). The cDNA samples were diluted at 1:10 and amplified with Sso Fast EvaGreen Supermix through the Cfx96-360 Bio-Rad Real-Time system (Bio-Rad). Genes and primers used are reported in Supporting Information 1: Table S2. The housekeeping gene *Gapdh* was chosen for normalization. The relative transcript level was determined using the *ΔΔ*Ct method.

### 2.8. Western Blot Analysis

Cells were plated in 100 mm petri dishes at a density of 4,000,000 cells/dish, allowed to acclimate for 24 h, and treated with Doxo (0.5 μM and 1 μM) with or without 125 µM RED for 48 h.

For total protein extraction, cells were lysed with RIPA buffer (50 mM Tris-HCl pH 8, 150 mM NaCl, 1% IGEPAL, 0.5% sodium deoxycholate, 0.1% SDS, 2 mM EDTA, 0.5 M NaF, 200 mM PMSF, 100 mM sodium orthovanadate, and 10% glycerol) supplemented with proteases inhibitors (Protease Inhibitor Cocktail, PIC, Sigma).

For nuclear and cytoplasmic protein extraction, cells were lysed with Lysis Buffer A (10 mM Tris-HCl pH 8, 150 mM NaCl, 1 mM EDTA, 1% IGEPAL, 10% Glycerol, and 0.5% PIC) for 5 min on ice and centrifuged for 10 min at 9300 × *g* at 4°C. The resulting supernatant was stored as cytoplasmic fraction. The pellet was washed three times with 800 μL of Lysis Buffer A (centrifuged each time for 5 min at 9300 × *g* at 4°C), resuspended in 30 μL of Lysis Buffer B (50 mM Tris-HCl pH 6.8, 2% SDS in distilled water, and 0.5% PIC), and subjected to cyclic freezing in dry ice and boiling at 100°C for 5 min for at least four times. After 10 min of centrifugation at 9300 × *g* 4°C, the supernatant was collected as the nuclear fraction. Proteins were quantified with Bio-Rad Protein Assay (500–0006, Bio-Rad) diluted 1:5 using a BSA standard curve and absorbance was measured at 590 nm using the Eppendorf BioSpectrometer basic (Eppendorf). The purity of the two collected fractions was verified using α-tubulin or Vinculin and Histone H4 (H4) as cellular and nuclear markers, respectively.

For each sample, at least 20 μg of proteins underwent SDS-PAGE, followed by transfer onto nitrocellulose membranes. Primary and secondary antibodies used are listed in Supporting Information 1: Table S3. Bands were detected using Immobilon Western chemiluminescent HRP substrate (Millipore) and the ChemiDoc Imaging System (Bio-Rad). Band intensity was calculated using ImageJ software and normalized against loading control proteins: Vinculin for AMPK and p53, H4 for nuclear fraction and α-tubulin for cytoplasmic fraction, FGF21 and SIRT1.

### 2.9. Enzyme-Linked Immunosorbent Assay (ELISA)

To quantify the amount of secreted FGF21, cells were seeded in a 48-well plate at a concentration of 150,000 cells/well for 24 h and treated with different concentrations of Doxo (0.5 μM and 1 μM) with or without 125 µM RED for 48 h. Supernatant samples were analyzed using the FGF21 Duo-Set ELISA kit (R&D Systems) following the supplier's instructions. For each well, total proteins were also extracted and quantified using the Bio-Rad Protein Assay. The optical density was analyzed at a wavelength of 450 nm using a microplate reader (Tecan Infinite F200PRO). The pg/mL obtained from the ELISA assay were normalized on the µg of total protein in each well.

### 2.10. Topoisomerase 2β (Top2β) Decatenation Assay

The activity of Top2β was evaluated using the kinetoplast DNA (kDNA) as substrate (Topo II beta decatenation assay kit, Inspiralis) following the supplier's instruction. Samples were loaded onto a 1% agarose gel stained with 0.5 µg/mL ethidium bromide and documented using a UV system combined with a camera (GBC).

### 2.11. Rapid Approach to DNA Adducts Recovery (RADAR) Assay for Detection of Top2β Covalent Complexes

To detect Top2β-DNA covalent complex in cells treated with Doxo and/or RED, we performed the RADAR assay following the protocol described by Kiianitsa and Maizels [19] with some modifications. In brief, HL-1 cells were seeded in a 100 mm petri dish at a density of 1,000,000 cells/dish, incubated at 37°C for 24 h and treated with 0.5 µM Doxo with or without 125 µM RED for 48 h. Cells were then washed twice with PBS, incubated for 1 h with 4% formaldehyde solution, washed again with PBS, and lysed in 1 mL of lysis buffer (5 M guanidinium isothiocyanate, 10 mM Tris-HCl pH 6.5, 20 mM EDTA, 4% Triton X-100, 1% sarkosyl, and 1% dithiothreitol). The cell lysate was mixed with 500 µL of 100% ethanol, vortexed thoroughly, and incubated for 5 min at −20°C. Samples were centrifuged at 21,000 × *g* for 30 min; the pellets were washed three times in 1 mL of 75% ethanol and centrifuged for 10 min at 21,000 × *g*. The DNA pellet was then dissolved in 500 µL of freshly prepared 8 mM NaOH, incubated in a water bath at 65°C for 5 min, and allowed to cool. DNA concentration was evaluated using a NanoDrop One/OneC (Thermo Fisher Scientific) at 260 nm wavelength. For each sample, 2 µg of DNA were diluted in 200 µL of 25 mM NaPO_4_ pH 6.5. Samples were loaded in duplicate onto a nitrocellulose membrane using a slot-blot apparatus as previously described [20]. The membrane was incubated with Top2β primary antibody (sc-365071, Santa Cruz, Dallas, Texas, USA, diluted 1:1000 in 1% BSA/TBST), H4 primary antibody (2935, Cell Signaling Technology, Danvers, MA, USA, diluted 1:1000 in 1% BSA/TBST) as a loading control, and the anti-mouse secondary antibody (ab205719, Abcam, diluted 1:5000 in 1% BSA/TBST). The membrane was developed with Immobilon Western chemiluminescent HRP substrate (Millipore); the signal was captured using the ChemiDoc Imaging System (Bio-Rad).

### 2.12. NAD^+^/NADH Quantification Assay

Cells were plated in 100 mm petri dishes at a density of 8,000,000 cells/dish, allowed to acclimate for 24 h, and treated with Doxo (0.5 μM and 1 μM) with or without 125 µM RED for 48 h. Extraction was performed using NAD^+^/NADH assay kit (ab65348, Abcam) following the supplier's instructions. Optical density was measured at 450 nm wavelength using a microplate reader (EnSight Perkin Elmer), with multiple readings over 3 h. Data are presented as NAD^+^/NADH ratio in pmol/µL.

### 2.13. Statistical Analysis

Data are presented as mean ± SEM and were analyzed using GraphPad Prism 6 by two-way ANOVA followed by Tukey's or Sidak's multiple comparisons test, as reported in the figure legends. The number of independent biological experiments, technical replicates and animals/samples is also reported in the figure legends. *p*-values lower than 0.05 were considered statistically significant (*⁣*^*∗*^*p*  < 0.05, *⁣*^*∗∗*^*p*  < 0.01, *⁣*^*∗∗∗*^*p*  < 0.001, *⁣*^*∗∗∗∗*^*p*  < 0.0001).

## 3. Results

### 3.1. RED Protected Cardiomyocytes Against Doxo-Induced Cell Death

**Figure 1:**
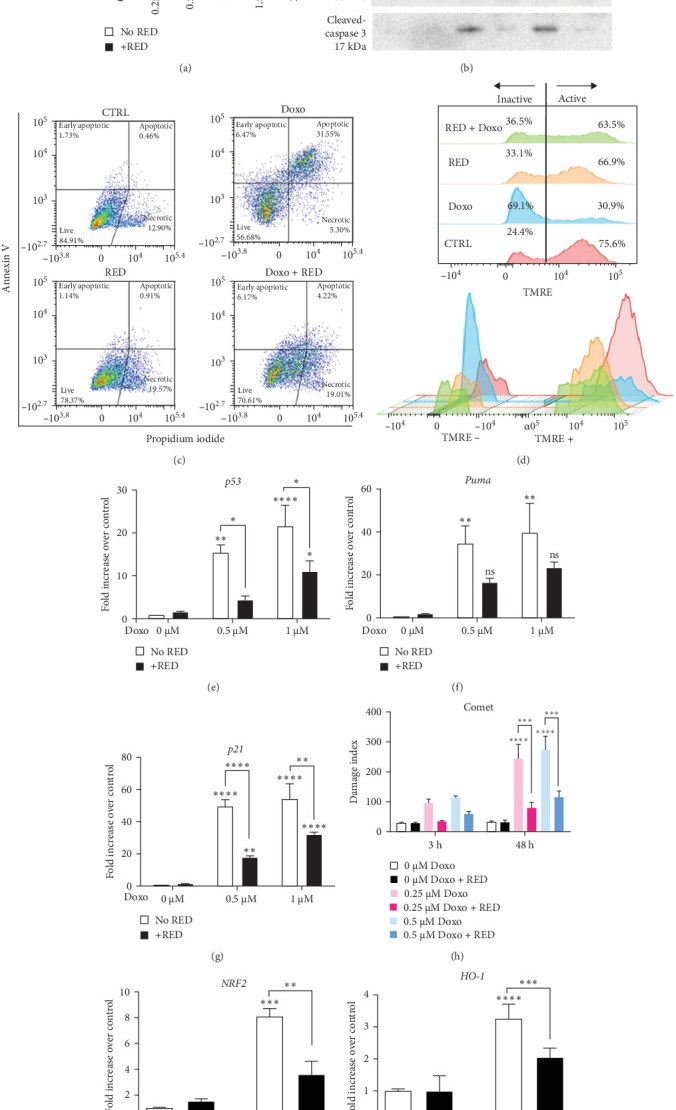
RED prevented Doxo-induced cardiotoxicity by inhibiting apoptosis, cell cycle arrest, and DNA damage. (a) MTT assay on HL-1 cells treated with increasing concentrations of Doxo with or without 125 µM RED. Data are presented as mean ± SEM of three technical replicates per condition, from three independent biological experiments and were analysed using two-way ANOVA followed by Sidak's multiple comparisons test. (b) Western blot analysis of cleaved-caspase 3 on HL-1 cells treated with 0.5 or 1 µM Doxo with or without 125 µM RED. Results from one of three independent biological experiments are shown. (c) Flow cytometry analysis of Annexin V and propidium iodide (PI) staining of apoptotic cells following 24 h treatment with 0.5 μM Doxo treatment with or without 48 h 125 µM RED. (d) Flow cytometry analysis of mitochondria membrane potential using tetramethylrhodamine ethyl ester (TMRE). Histograms show untreated cells (red), 0.5 µM Doxo-treated cells (blue), and 0.5 µM Doxo + 125 µM RED (green). Representative data from two independent experiments are shown. (e) qPCR analysis of *p53*, (f) *Puma*, and (g) *p21* genes after 48 h treatment with 0.5 and 1 µM Doxo with or without 125 µM RED. Data are expressed as mean ± SEM of three technical replicates, from four independent biological experiments and were analyzed using two-way ANOVA followed by Sidak's multiple comparisons test. (h) Comet assay on HL-1 cells after 3 and 48 h treatment with 0.25 and 0.5 µM Doxo with or without 125 µM RED. Data are expressed as mean ± SEM from three independent biological experiments and were analyzed using two-way ANOVA followed by Sidak's multiple comparisons test. (i) qPCR analysis of *NRF2* and (j) *HO-1* genes after 48 h treatment with 0.25 µM Doxo with or without 125 µM RED. Data are expressed as mean ± SEM of three technical replicates, from two independent biological experiments and were analyzed using two-way ANOVA followed by Tukey's multiple comparisons test. *⁣*^*∗*^*p*  < 0.05, *⁣*^*∗∗*^*p*  < 0.01, *⁣*^*∗∗∗*^*p*  < 0.001, and *⁣*^*∗∗∗∗*^*p*  < 0.0001 indicate significant differences versus 0 µM Doxo and between indicated pairs.

Despite being an effective chemotherapeutic drug, Doxo causes cumulative and dose-dependent cardiotoxicity and death of cardiomyocytes through several mechanisms that ultimately lead to apoptosis [21]. We investigated the role of purple corn extract (RED) in the protection against Doxo-induced cytotoxicity in HL-1 cardiomyocytes. As demonstrated by the MTT assay, RED cotreatment significantly improved cell viability up to 1 μM Doxo (Figure 1a). Western blot analysis showed a dramatic decrease in cleaved-caspase 3 signal when Doxo was used in combination with RED (Figure 1b), while Annexin V-PI staining confirmed that the presence of RED in cardiomyocytes exposed to Doxo dramatically reduced the percentage of apoptotic cells from 35.8% down to 4.08%, without affecting the percentage of necrotic cells (Figure 1c). This evidence demonstrates that RED administration helps to prevent Doxo-induced apoptosis.

Mitochondrial transmembrane potential is a key parameter of mitochondrial function. Its loss follows the opening of the mitochondrial permeability transition pores, resulting in the release of cytochrome C and activation of the apoptotic cascade [22]. Depolarized or inactive mitochondria show decreased membrane potential and fail to sequester TMRE [23]. When TMRE was used to label active mitochondria in HL-1 cardiomyocytes, we observed a dramatic increase in the percentage of inactive mitochondria upon Doxo treatment (69.1%), which was prevented by pre-treatment with RED (36,5% inactive mitochondria; Figure 1d), confirming the RED protective effect against apoptosis. RED also reduced the upregulation of genes involved in apoptosis (*p53* and *Puma*, Figure 1e,f) and cell cycle arrest (*p21*, Figure 1g), and limited Doxo-induced DNA damage, reducing the damage index by 68% and 58% for 0.25 µM Doxo and 0.5 µM Doxo, respectively (Figure 1h). Interestingly, RED significantly attenuated the Doxo-induced upregulation of the *NRF2* gene and its target *HO-1* gene (Figure 1i,j), indicating that RED may confer cytoprotective effects against Doxo-induced oxidative stress in HL-1 cardiomyocytes, as suggested by the reduced activation of the endogenous antioxidant response. Overall, these findings suggest that RED might prevent Doxo-induced apoptosis by limiting DNA damage and cell cycle arrest in cardiomyocytes.

### 3.2. RED Prevented Doxo-Induced Increase of AMPK Activity

**Figure 2:**
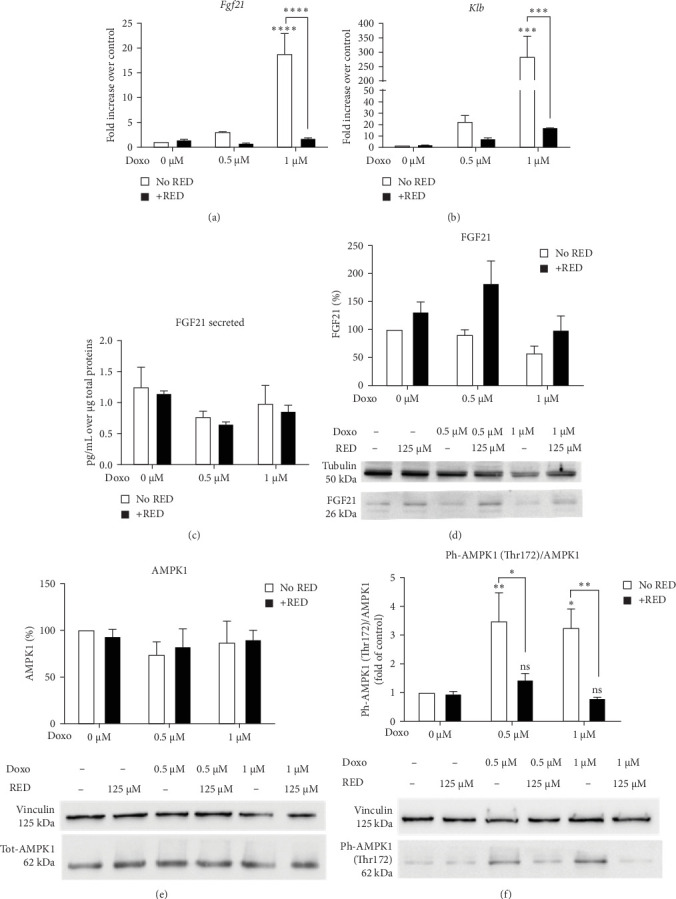
RED prevented Doxo-induced increase of AMPK activity. (a, b) qPCR analysis of *Fgf21* and its receptor *Klb* gene expression. Data are expressed as mean ± SEM of three technical replicates from three independent biological experiments and were analyzed using two-way ANOVA followed by Tukey's multiple comparisons test. (c) ELISA analysis of secreted FGF21. Data are expressed as mean ± SEM of two technical replicates from two independent biological experiments and were analyzed using two-way ANOVA followed by Tukey's multiple comparisons test. (d) Western blot analysis of FGF21 protein expression in HL-1 cells treated for 48 h with 0.5 or 1 µM Doxo with or without RED. Data are presented as mean ± SEM of six independent biological experiments and were analyzed using two-way ANOVA followed by Sidak's multiple comparisons test. (e) Western blot analysis of total AMPK1 protein and (f) phosphorylated AMPK1 at Threonine 172. Results on phosphorylated AMPK1 are presented as the ratio over total AMPK1. Data are presented as mean ± SEM from two independent biological experiments, respectively, and were analyzed using two-way ANOVA followed by Sidak's multiple comparisons test. *⁣*^*∗*^*p*  < 0.05, *⁣*^*∗∗*^*p*  < 0.01, *⁣*^*∗∗∗*^*p*  < 0.001, and *⁣*^*∗∗∗∗*^*p*  < 0, 0001 indicate significant differences versus 0 µM Doxo and between indicated pairs.

Despite being weakly expressed in the heart, FGF21 contributes to several physiological processes, mainly via its action as metabolic regulator and cardioprotective agent [24]. Once secreted, FGF21 can act in an autocrine manner thanks to the transmembrane β-Klotho (Klb) coreceptor, expressed at low levels in cardiomyocytes [6]. Our results show that the transcript levels of the *Fgf21* gene and its coreceptor *Klb* gene were upregulated by Doxo treatment and restored to control levels by RED (Figure 2a,b). The intracellular protein level of FGF21 was not significantly affected by Doxo or RED (Figure 2d). Furthermore, neither Doxo nor RED appeared to affect FGF21 secretion (Figure 2c).

Compelling evidence showed that endocrine FGF21 signaling activates the AMPK pathway, either directly through FGFR1/β-Klotho or indirectly through adiponectin and corticosteroids [24, 25]. In our setting, Doxo promoted the activation of AMPK, as confirmed by phosphorylation at AMPK-Thr172, whereas RED co-treatment restored AMPK activity to control levels (Figure 2e,f).

### 3.3. Doxo and RED Effects on SIRT1 and p53

**Figure 3:**
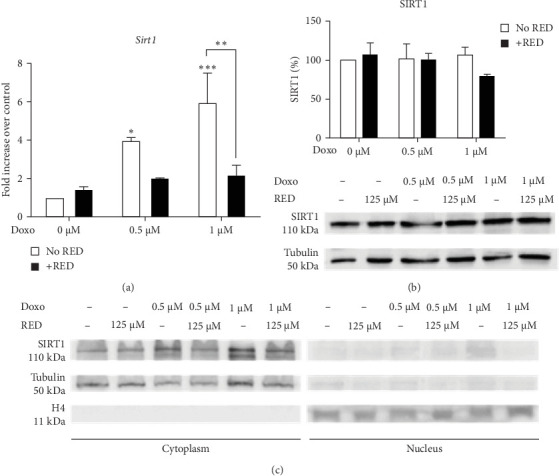
*Sirt1* expression and subcellular localization. (a) qPCR analysis of *Sirt1* gene expression. Data are expressed as mean ± SEM of three technical replicates from three independent biological experiments and were analyzed using two-way ANOVA followed by Tukey's multiple comparisons test. (b) Western blot analysis of SIRT1 protein expression and (c) SIRT1 subcellular localization in HL-1 cells treated for 48 h with 0.5 or 1 µM Doxo with or without RED. Data in Subpart (b) are presented as mean ± SEM of four independent biological experiments and were analyzed using two-way ANOVA followed by Sidak's multiple comparisons test. Subpart (c) shows representative blots from two independent biological experiments. *⁣*^*∗*^*p*  < 0.05, *⁣*^*∗∗*^*p*  < 0.01, and *⁣*^*∗∗∗*^*p*  < 0.001 indicate significant differences versus 0 µM Doxo and between indicated pairs.

Once activated, AMPK activates in turn SIRT1 through phosphorylation and detachment from its inhibitor DBC1 [26]. Upon activation, SIRT1 generally translocate into the nucleus, activating an antioxidant response; however, it has been reported that in differentiated muscle cell lines like C2C12, SIRT1 remains and acts in the cytoplasm [27]. In our setting, the *Sirt1* gene was upregulated after Doxo treatment and its expression was reduced by RED treatment (Figure 3a), although no effects were seen on SIRT1 protein levels (Figure 3b). At both at 24 h (data not shown) and 48 h, SIRT1 localized in the cytoplasm and did not translocate into the nucleus following Doxo or RED treatment (Figure 3c).

**Figure 4:**
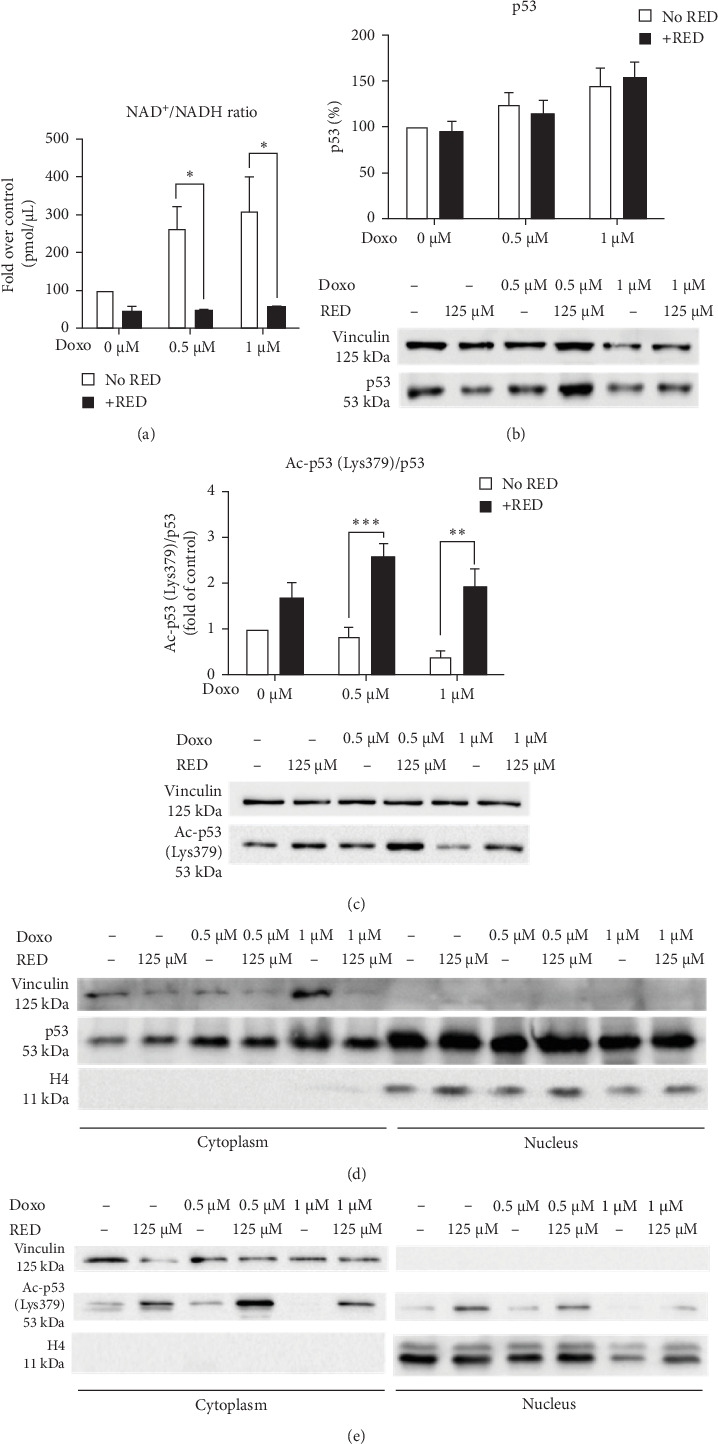
RED increased the p53 acetylation at Lys379. (a) NAD^+^/NADH ratio quantification. Data are presented as mean ± SEM of three independent biological experiments and were analyzed using two-way ANOVA followed by Sidak's multiple comparisons test. (b) Western blot analysis on total p53, (c) acetylated p53 at Lysine 379, and (d, e) their subcellular localization. Acetylated p53 levels are reported as the ratio over total p53. Data in Subparts (b,c) are presented as mean ± SEM of three and four independent biological experiments, respectively, and were analyzed using two-way ANOVA followed by Sidak's multiple comparisons test. Subparts (d, e) show representative blots of two independent biological experiments. *⁣*^*∗*^*p*  < 0.05, *⁣*^*∗∗*^*p*  < 0.01, and *⁣*^*∗∗∗*^*p*  < 0.001 indicate significant differences versus 0 µM Doxo and between indicated pairs.

Since SIRT1 is a NAD-dependent deacetylase activated by a high NAD^+^/NADH ratio [28], we investigated this ratio to determine whether Doxo and RED could affect SIRT1 activity. Our results show that Doxo induced an increase in the NAD^+^/NADH ratio, suggesting that SIRT1 activity was increased by Doxo and maintained at control levels by RED treatment (Figure 4a). Another indirect indicator of SIRT1 activity is the deacetylation of its targets, such as SIRT1-dependent deacetylation of p53 at Lys382 (Lys379 in mouse). p53 acetylation is known to induce the expression of *p21* and *Puma* genes, cell cycle arrest and DNA repair upon DNA damage [9], which is one of the causes of Doxo-induced cardiotoxicity. Our results show that p53 total protein levels were not significantly altered by Doxo and/or RED (Figure 4b), whereas acetylation of p53 at Lys379 was reduced by Doxo treatment and significantly increased by RED extract (Figure 4c). Concerning p53 subcellular localization, p53 localized in both the cytoplasm and in the nucleus (Figure 4d), but acetylated p53 at Lys379 appeared more prevalent in the cytoplasm (Figure 4e), suggesting a suppression of p53 transcriptional activity by RED treatment.

### 3.4. Fgf21 and Sirt1 Silencing

**Figure 5:**
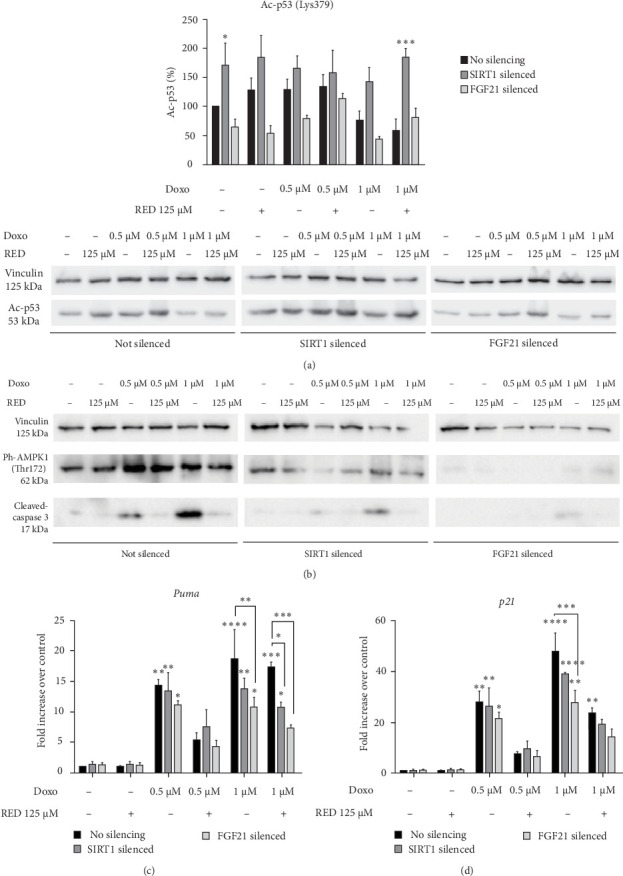
Effect of *Fgf21* and *Sirt1* silencing on p53 acetylation and AMPK activity. Western blot analysis of the effects of *Sirt1* and *Fgf21* silencing on (a) Ac-p53(Lys379), (b) Ph-AMPK1(Thr172), and cleaved-caspase 3 in HL-1 cells treated with 0.5 or 1 µM Doxo with or without 125 µM RED. Data in Subpart (a) are presented as mean ± SEM of three independent biological experiments and were analyzed using two-way ANOVA followed by Sidak's multiple comparisons test. Representative blots from one of the three independent biological replicates are shown. Subpart (b) shows representative blots of two independent biological experiments. qPCR analysis of the effects of *Sirt1* and *Fgf21* silencing on (c) *Puma* and (d) *p21* gene expression in HL-1 cells treated with 0.5 or 1 µM Doxo with or without 125 µM RED. Data are expressed as mean ± SEM of three technical replicates per condition, from two independent biological experiments and were analyzed using two-way ANOVA followed by Tukey's multiple comparisons test. *⁣*^*∗*^*p*  < 0.05, *⁣*^*∗∗*^*p*  < 0.01, *⁣*^*∗∗∗*^*p*  < 0.001, and *⁣*^*∗∗∗∗*^*p*  < 0.0001 indicate significant differences versus 0 µM Doxo and between indicated pairs.

To further analyze the role of FGF21 and SIRT1 in Doxo-mediated cardiotoxicity, we silenced *Fgf21* or *Sirt1* with gene-specific siRNAs in Doxo-treated HL-1 cells in the presence or absence of RED extract (Supporting Information 2: Figure S1). *Fgf21* silencing reduced *Fgf21* transcript and secreted FGF21 protein levels (Supporting Information 2: Figure S1a,b), but not SIRT1 protein levels (Supporting Information 2: Figure S1d,e). Similarly, *Sirt1* silencing significantly reduced both *Sirt1* transcript and SIRT1 protein levels (Supporting Information 2: Figure S1c–e), but not secreted FGF21 protein levels (Supporting Information 2: Figure S1b), indicating that the silencing of *Sirt1* and *Fgf21* was gene-specific. Our results show that *Fgf21* silencing greatly reduced AMPK1 phosphorylation and caspase 3 activation (Figure 5b), as well as the transcript levels of *p21* and *Puma* genes upon 1 μM Doxo (Figure 5c,d). Furthermore, a slight reduction in acetylated p53 levels was observed compared to nonsilenced samples, albeit not statistically significant (Figure 5a). *Sirt1* silencing partially reduced AMPK1 phosphorylation at Thr172 and caspase 3 activation (Figure 5b) and resulted in a general increase in p53 acetylation at Lys379 (Figure 5a), thus, confirming the correlation between increased SIRT1 activity and decreased p53 acetylation. Consistent with this, *Sirt1* silencing also led to a reduction of *Puma* and *p21* transcript levels upon 1 μM Doxo (Figure 5c,d).

### 3.5. C3G Prevented ROS Accumulation and Cytotoxicity in Primary Cardiomyocytes

**Figure 6:**
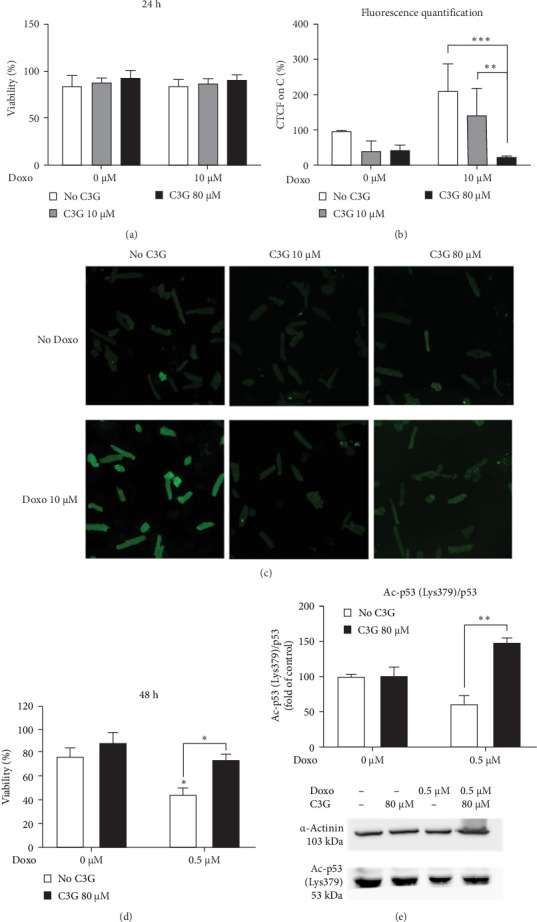
Effect of cyanidin 3-glucoside on ROS accumulation and cytotoxicity in primary cardiomyocytes. (a) Viability of primary cardiomyocytes at 24 h of treatment. Results presented as the percentage of living cells at 24 h divided by the number of living cells at 0 h, based on cell shape. Data are expressed as mean ± SEM (*n* = 3 mice from three independent experiments). (b, c) Confocal spinning disk microscopy images (20x) and relative fluorescence quantification. For each treatment, 4–8 cells were considered. Results are expressed as mean ± SEM (*n* = 3 mice from three independent experiments); *⁣*^*∗∗*^*p*  < 0.01 and *⁣*^*∗∗∗*^*p*  < 0.001 indicate significant differences (two-way ANOVA followed by Tukey's multiple comparisons test) compared to 0 μM and between the indicated pairs. These results were obtained on mouse primary cardiomyocytes pretreated with 10 or 80 µM C3G for 2 h, followed by treatment with 10 µM Doxo with or without 10 or 80 µM C3G for 24 h. (d) Viability of primary cardiomyocytes after 48 h of treatment. Results are presented as the percentage of living cells at 48 h divided for living cells at 0 h, the count was based on cell shape. (e) Western blot analysis of p53 acetylation at Lys379. These results were obtained in mouse primary cardiomyocytes treated with 0.5 µM Doxo with or without 80 µM C3G for 48 h. Results in Subpart (d) are expressed as mean ± SEM (*n* = 7 mice from seven independent experiments), whereas results in Subpart (e) are expressed as mean ± SEM (*n* = 4 mice from four independent experiments); *⁣*^*∗*^*p*  < 0.05 and *⁣*^*∗∗*^*p*  < 0.01 indicate significant differences (two-way ANOVA followed by Tukey's multiple comparisons test) compared to 0 μM and between the indicated pairs.

To confirm that the RED-induced cardioprotective activity may be mediated by C3G, the main ACN present in purple corn [14], and by p53 acetylation, we verified whether C3G can prevent Doxo-induced ROS accumulation and cytotoxicity in murine primary cardiomyocytes. First, cells were pretreated for 2 h with C3G (10 or 80 µM) and then, challenged with 10 µM Doxo for 24 h. In this setting, viability of primary cardiomyocytes was not altered either by 10 µM Doxo or C3G (Figure 6a). However, the intracellular ROS levels, measured using DCF, increased upon 10 µM Doxo treatment, and were significantly decreased by 80 µM C3G (Figure 6b,c). Second, we evaluated the viability of primary cardiomyocytes pretreated for 2 h with 80 µM C3G and then, challenged for 48 h with 0.5 µM Doxo, the same Doxo concentration used for HL-1 cells. Our results show that 0.5 µM Doxo reduced cell viability at 30%, whereas the cotreatment with 80 µM C3G restored viability to the control level (Figure 6d). Furthermore, p53 acetylation at Lys379 was reduced by 0.5 μM Doxo and increased by cotreatment with 80 µM C3G (Figure 6e), thus, confirming the p53 acetylation pattern observed in HL-1 cells.

### 3.6. Doxo and RED Effects on Top2β

**Figure 7:**
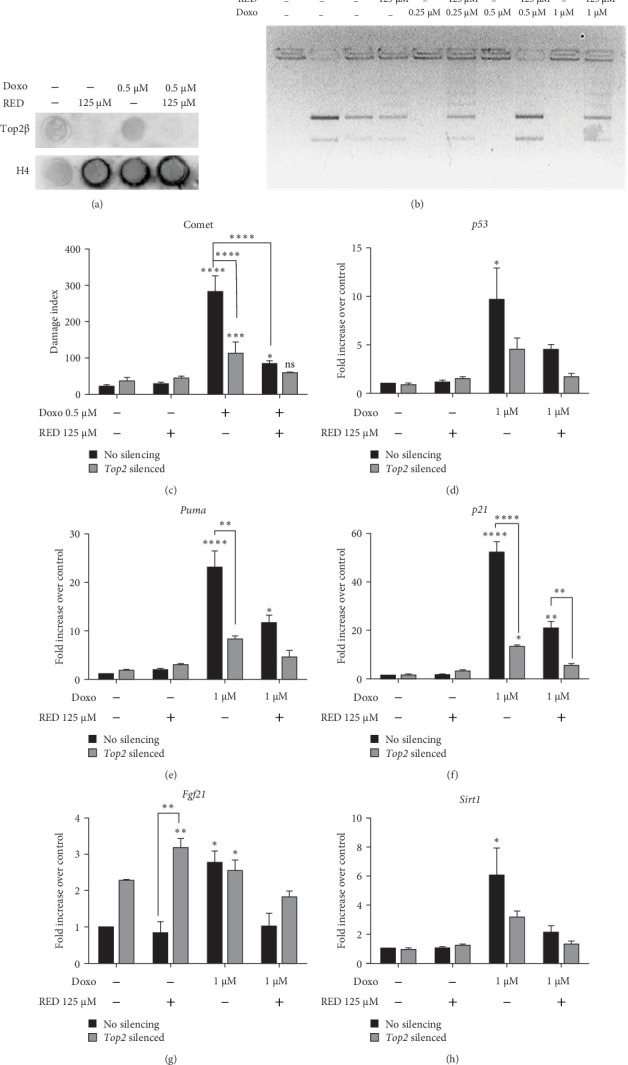
Doxo and RED effects on Topoisomerase 2β. (a) RADAR assay on HL-1 cells treated for 48 h with 0.5 μM Doxo with or without 125 µM RED. (b) Decatenation assay performed on cell extracts from HL-1 cells treated for 48 h with 0.25, 0.5, or 1 µM Doxo with or without 125 µM RED. One representative blot from three independent biological experiments is shown in Subparts (a, b). (c) Effect of *Top2* silencing on DNA damage in HL-1 cells treated for 48 h with 0.5 µM Doxo with or without 125 µM RED analyzed by comet assay. Data are expressed as mean ± SEM from three independent biological experiments and were analyzed using two-way ANOVA followed by Sidak's multiple comparisons test. (d–h) qPCR analysis of the effects of *Top2* silencing on *p53*, *Puma*, *p21*, *Fgf21*, and *Sirt1* gene expression in HL-1 cells treated for 48 h with 1 µM Doxo ± 125 µM RED. Data are expressed as mean ± SEM of three technical replicates per condition from two independent biological experiments and were analyzed using two-way ANOVA followed by Tukey's multiple comparisons test. *⁣*^*∗*^*p*  < 0.05, *⁣*^*∗∗*^*p*  < 0.01, *⁣*^*∗∗∗*^*p*  < 0.001, and *⁣*^*∗∗∗∗*^*p*  < 0.0001 indicate significant differences versus 0 µM Doxo and between indicated pairs.

It is known that Doxo inactivates Top2β by trapping it on the DNA in a ternary complex [29], and RED restores Top2β activity by preventing its permanent binding to DNA (Figure 7a,b). The RADAR assay detects the covalent binding of Top2β to DNA through immunodetection of protein-DNA adducts. Top2β was immunodetected in cells treated with Doxo alone, but not in those treated with Doxo and RED, suggesting that RED reduced the stabilization of the covalent bond of Top2β to DNA caused by Doxo (Figure 7a).

The result was confirmed by the Top2β decatenation assay (Figure 7b). The assay tests Top2β activity through its ability to resolve the catenated kDNA from *Crithidia fasciculata*, consisting of a network of many DNA minicircles (2.5 Kb). In cells cotreated with Doxo and RED, decatenated products were visible in the bottom part of the gel, while in cells treated with Doxo alone, the kDNA fluorescence was visible just below the well because the kDNA fails to enter the gel. This means that Top2β retained its activity in the presence of RED (Figure 7b).

The silencing of *Top2* greatly reduced the DNA damage induced by Doxo and the cotreatment with RED completely prevented Doxo-induced DNA damage (Figure 7c). Moreover, *Top2* silencing reduced the Doxo-induced upregulation of *p53*, *Puma*, and *p21* genes, and the concomitant administration of RED completely prevented their upregulation (Figure 7). *Top2* silencing also reduced *Sirt1* gene upregulation (Figure 7h), but surprisingly increased *Fgf21* gene expression, even in the presence of RED (Figure 7g).

## 4. Discussion

The aim of this study was to investigate the protective effect of RED extract against Doxo toxicity in HL-1 cardiomyocytes focusing on the AMPK/SIRT1/p53 pathway. Our previous studies demonstrated that dietary ACNs from purple corn can protect the heart against Doxo-induced cardiotoxicity in vivo and promote HL-1 cell viability, without reducing the antitumoral activity of Doxo in vitro [14]. Here, we confirmed that RED extract significantly reduced mitochondrial damage and improved HL-1 cell viability up to 1 µM Doxo. It is known that Doxo-induced cardiomyocyte apoptosis is p53-dependent, with the upregulation of *Puma* and *p21*, which trigger the intrinsic apoptotic pathway and cell cycle arrest, respectively [30, 31]. Consistent with this, we showed that *p53*, *Puma*, and *p21* transcript levels and cleaved-caspase 3 protein levels were increased by Doxo treatment and restored to control levels by RED cotreatment, thus, indicating that RED extract prevented Doxo-induced apoptosis in HL-1 cardiomyocytes. In addition, consistent with reduced DNA damage, RED was found to modulate the NRF2/HO-1 response pathway in HL-1 cardiomyocytes, suggesting a potential role in mitigating the oxidative damage induced by Doxo.

Recent studies reported that FGF21 exerts its protective role in cardiomyocytes by activating the AMPK/SIRT1/p53 pathway, and it can induce AMPK activation through phosphorylation [7, 32]. Conflicting results about the effects of Doxo on AMPK are reported in the literature. Some studies showed that Doxo exerts its cardiotoxic effect through AMPK inactivation, while others have reported AMPK activation [33–36]. The effect of Doxo on AMPK may depend on several parameters, such as Doxo dosage, exposure time, and the type of cell model used [37]. Our results showed that Doxo induced AMPK activation via Thr172 phosphorylation and RED restored AMPK activity to control levels. *Fgf21* and its receptor *Klb* genes were upregulated by Doxo and maintained at control levels by RED. No effects on total and secreted FGF21 protein levels were observed, but *Ffg21* silencing dampened AMPK phosphorylation and caspase 3 cleavage, suggesting that AMPK phosphorylation is mainly controlled by FGF21 and is responsible for apoptosis in HL-1 cells. Furthermore, the simultaneous silencing of *Fgf21* and RED cotreatment prevented the Doxo-induced AMPK activation and cleaved-caspase 3 formation.

SIRT1 is a NAD-dependent deacetylase involved in many cellular processes, including ROS scavenging, cell death/survival signaling, and aging. It has recently been pointed out that it may play a role in cardioprotection, since its absence causes severe damages in developing hearts [5]. In our model, Doxo increased *Sirt1* gene expression but had no effect on its protein levels. RED effectively counteracted the Doxo-induced *Sirt1* gene upregulation, without affecting its protein level. The silencing of *Top2*, with the subsequent reduction in DNA damage, lowered Doxo-induced *Sirt1* gene upregulation, in line with SIRT1 role in the DNA damage response [38]. Some studies have described that under stress or heart failure conditions, SIRT1 translocates into the nucleus and activates an antioxidant response [39, 40]. However, it has also been reported that in embryonic cardiomyocytes, SIRT1 is more prevalent in the nucleus, while in adult cardiomyocytes it is predominantly found in the cytoplasm. Our analysis of the subcellular localization of SIRT1 showed that it remains in the cytoplasm under all treatment conditions. HL-1 cells are differentiated cardiomyocytes, and this could explain the cytoplasmic localization of SIRT1. Consistent with this, in C2C12 myoblasts, SIRT1 is expressed only in the nucleus, whereas after differentiation it is present only in the cytoplasm in about 40% of the cells [27].

It has been shown that SIRT1 activity on p53 is AMPK-mediated: the binding between SIRT1 and one of its regulators, DBC1, depends on AMPK, which promotes SIRT1-DBC1 dissociation and SIRT1 activation by direct phosphorylation [9]. Moreover, AMPK can indirectly activate SIRT1 by modifying NAD^+^/NADH ratio [41]. Our results showed that increased phosphorylation of AMPK induced by Doxo was correlated with an increased NAD^+^/NADH ratio and subsequent SIRT1 activation, as measured by p53 Lys379 deacetylation. p53 acetylation is known to increase its transactivation ability, inducing the expression of *p21* and *Puma*, which leads to cell cycle arrest, providing time to the cells to repair DNA. SIRT1 controls p53 deacetylation at Lys379. Many studies report that p53 deacetylation at Lys379 leads to reduced *p21* and *Puma* transactivation [8, 38, 42]. Our data seem to contrast the literature and the common idea that Lys379 acetylation increases p53 transcription factor activity. However, a study by Brochier et al. [43] suggests that the p53 Lys379 acetylation can have opposite effects in different cell types. In neurons, p53 acetylation decreases its DNA binding affinity at both *Puma* and *p21* response elements, while in tumor cell lines, acetylated p53 displays enhanced *Puma* promoter transactivation and pro-apoptotic activity [43]. Our results indicated that RED treatment increased p53 Lys379 acetylation while downregulating *p21* and *Puma* gene expression and caspase 3 activation, suggesting that the increase in p53 acetylation might have a protective role in HL-1 cardiomyocytes. Furthermore, p53 was localized equally in both the cytoplasm and the nucleus under all tested conditions, whereas the acetylated form was mainly present in the cytoplasm, suggesting a suppression of p53 transcriptional activity upon RED cotreatment. We then confirmed in mouse ventricular primary cardiomyocytes that ACNs increased p53 acetylation and protected against Doxo-induced ROS accumulation and loss of cell viability, thus, proving to be effective in counteracting Doxo-induced apoptosis. Future ChIP-qPCR studies will be necessary to directly assess p53 binding to *p21* and *Puma* promoters, thereby clarifying whether RED modulates p53 transcriptional activity through altered DNA binding.

In agreement with previous results, *Sirt1* silencing reduced AMPK activation and increased p53 acetylation. Conversely, *Sirt1* silencing did not affect FGF21 protein secretion, but it decreased cleaved-caspase 3 production. RED treatment was more effective than *Sirt1* silencing in reducing cleaved-caspase 3, suggesting that RED does not exert its protective effect solely through SIRT1. *Fgf1* silencing also downregulated *p21* and *Puma*, which were further downregulated by RED cotreatment together with *Fgf21* silencing.

Our previous studies, together with these findings, suggest the potential use of dietary ACNs from purple corn in the prevention of cardiotoxicity in patients undergoing anthracycline chemotherapy. In fact, we previously demonstrated that in mice fed an ACN-rich diet from purple corn (red diet [RD]), which provided about 36 mg/kg body weight/day of C3G, the typical Doxo-induced cardiac alterations (i.e., disorganized myofibrils, mitochondrial fragmentation and degradation, and defects in sarcolemma junctions) were prevented compared to mice fed an ACN-free diet (yellow diet [YD]). In addition, this ACN dose resulted in a significant reduction in short and mid-term Doxo-induced mortality in mice [14]. The human equivalent dose (HED) corresponding to the ACN daily dose administered to Doxo-treated mice is approximately 175 mg [44], which is comparable to the dose administered to Chron's disease patients undergoing Infliximab therapy, using an orally administrable RED extract obtained from purple corn cobs (i.e., 125 mg/day), which showed high palatability, an optimal safety profile and significant anti-inflammatory properties [45, 46].

Other studies have demonstrated that dietary ACNs at comparable doses also improve hemodynamic function or ejection fraction in animal models. In fact, the administration of a blueberry extract (i.e., 20–80 mg/kg body weight) to rats treated with cyclophosphamide, another cardiotoxic chemotherapeutic drug, significantly attenuated increases in mean arterial blood pressure, decreases of left ventricular (LV) ejection fraction as well as LV hypertrophy and fibrosis [47]. Furthermore, dietary ACNs from bilberry were shown to improve Doxo-induced ECG abnormalities, such as bradycardia, ST segment depression, and prolongation of both ST and QT intervals [13].

## 5. Conclusions

Our study highlights the beneficial effects of the RED extract in counteracting the cardiotoxicity induced by Doxo. Our results suggest that in HL-1 cardiomyocytes Doxo induces AMPK activation, leading to an increase in NAD^+^/NADH ratio and SIRT1 activation. Once active, SIRT1 deacetylates p53 at Lys379, which then becomes more localized in the nucleus, where it transactivates its target genes, *Puma* and *p21*, leading to cell death. Conversely, RED prevents the activation of AMPK, reducing NAD^+^/NADH ratio and SIRT1 activation. As a consequence, p53 remains acetylated, thus, not activating *Puma* and *p21*, leading to reduced apoptosis and increased cardiomyocyte survival.

## Data Availability

The data used to support the findings are available from the corresponding author upon request.

## Supporting Information

Supporting Information 1 Table S1. Target RNA sequences and siRNAs used for gene silencing. Table S2. List of primers. Table S3. List of primary and secondary antibodies.

Supporting Information 2 Figure S1. Effect of *Fgf21* and *Sirt1* silencing on transcript and protein levels. qPCR analysis of the effects of *Fgf21* and *Sirt1* silencing on (a) *Fgf21* and (c) *Sirt1* transcript levels, respectively. Data are expressed as mean ± SEM of three technical replicates per condition from two independent biological experiments and were analyzed using two-way ANOVA followed by Tukey's multiple comparisons test. (b) ELISA assay showing the effect of *Fgf21* and *Sirt1* silencing on FGF21 secretion. Data are presented as mean ± SEM of two technical replicates per condition from two independent biological experiments and were analyzed using two-way ANOVA followed by Tukey's multiple comparisons test. (d, e) Western blot analysis of the effects of *Fgf21* and *Sirt1* silencing on SIRT1 in HL-1 cells treated with 0.5 or 1 µM Doxo with or without 125 µM RED. Data in Subpart (d) are presented as mean ± SEM of three independent biological experiments and were analyzed using two-way ANOVA followed by Sidak's multiple comparisons test. Subpart (e) shows a representative blot from one of the three independent biological replicates. *⁣*^*∗*^*p* < 0.05, *⁣*^*∗∗*^*p* < 0.01, *⁣*^*∗∗∗*^*p* < 0.001, and *⁣*^*∗∗∗∗*^*p* < 0.0001 indicate significant differences versus 0 µM Doxo and between indicated pairs.
