# Supplementary material for: Purple Corn Extract Prevents Doxo-Induced Cardiotoxicity by Counteracting AMPK Activation and p53 Acetylation in HL-1 and Primary Cardiomyocytes
Source: Oxid Med Cell Longev. 2025 Sep 18;2025:7786043. doi: 10.1155/omcl/7786043 (PMC12463520; doi:10.1155/omcl/7786043)
Supplement: Supporting Information 1 — Table S1. Target RNA sequences and siRNAs used for gene silencing. Table S2. List of primers. Table S3. List of primary and secondary antibodies. [file 7786043.f1.pdf]

Supplementary Tables

Table S1. Target RNA sequences and siRNAs used for gene silencing

**Gene**	**Product name**	**Target sequence**
SIRT1	Sirt1-7, SI02717141, 20110624	CAGATTGTTATTAATATCCTT
SIRT1	Sirt1_8, SI02741984, 20110624	GAGGTTGTTAATGAAGCTATA
FGF21	Fgf21_2, SI01002414, 201701200041	TGGGTTTCCACTTATTTATTA
FGF21	Fgf21_4, SI01002428, 201811300071	CGGAGTCAGAACACAATTCCA
Top2α	Top2a_1, SI01453683, 201702070165	TTGGATCAACATGTCAATTAA
Top2α	Top2a_3, SI01453697, 20131218	CACTGTGAAGTTTGTCATAAA
Top2β	Top2b_5, SI02669023, 201712220220	TTCCTTTATGATGATAATCAA
Top2β	Top2b_6, SI02688945, 201712220221	GAGATAAATAATATTATTAAA

Table S2. List of primers

**Gene**	**Sequence**
BetaKlotho-F	5’-GTGACATGTACAACCGCACGA-3’
BetaKlotho-R	5’-TGCCCAGTCGCAATGTAAGG-3’
Fgf21-F	5’-CTGCTGGAGGACGGTTACAAT-3’
Fgf21-R	5’-GGAATCCTGCTTGGTCTTGG-3’
Gapdh-F	5’-AACTTTGGCATTGTGGAAGG-3’
Gapdh-R	5’-CACATTGGGGGTAGGAACAC-3’
HO-1-F	5’-CTCTCTTCTCTTGGGCCTCTAA-3’
HO-1-R	5’-TGTCAGGTATCTCCCTCCATTC-3’
Nrf2-F	5’-GTGATGTGAAATGCAGAAACACTT-3’
Nrf2-R	5’-TGGTGCCTAAGAAATTAACTTGAGA-3’
P21-F	5’-CGGTGGAACTTTGACTTCGTC-3’
P21-R	5’-GGAAGTACTGGGCCTCTTGT-3’
P53-F	5’-GCTTTGAGGTTCGTGTTTGTGC-3’
P53-R	5’-GCGGATCTTGAGGGTGAAATAC-3’
Puma-F	5’-AAGAAGAGCAGCATCGACAC-3’
Puma-R	5’-CTAGTTGGGCTCCATTTCTGG-3’
Sirt1-F	5’-GCTTGGAAGATGATGCTGAC-3’
Sirt1-R	5’-GTGCTCTGATTTGTCTGGTG-3’
Top2α-F	5’-GCCGACCTTCAACTACCTTC-3’
Top2α-R	5’-CAGCCAGATCTTCCTTCCAC-3’
Top2β-F	5’-TCGCCTTACGGGAGAAGAAT-3’
Top2β-R	5’-GCAGATGGGGTCAATGTCTC-3’

Table S3. List of primary and secondary antibodies

Primary antibody	*Host species*	*Supplier, code*
Acetyl-p53 (Lys379)	Rabbit	Cell Signaling, 2570
AMPKα	Rabbit	Cell Signaling, 2532
Cleaved-caspase 3	Rabbit	Cell Signaling, 9664
FGF21	Goat	R&D System, AF3057
Histone H4	Mouse	Cell Signaling, 2935
p53	Mouse	Cell Signaling, 2524 (1C12)
Phospho-AMPKα (Thr172)	Rabbit	Cell Signaling, 50081 (D4D6D)
SIRT1	Mouse	Abcam, ab110304
Tubulinα	Mouse	Sigma, T6074
Vinculin	Rabbit	GeneTex, GTX109749
Secondary antibody	***Buffer***	***Supplier, code***
Anti-mouse	1% BSA	Abcam, ab205719
Anti-goat	1% BSA	Biorad, 172-1034
Anti-rabbit	5% Milk	Jackson ImmunoResearch, 111035003
