# Supplementary figures and images for: Purple Corn Extract Prevents Doxo-Induced Cardiotoxicity by Counteracting AMPK Activation and p53 Acetylation in HL-1 and Primary Cardiomyocytes
Source: Oxid Med Cell Longev. 2025 Sep 18;2025:7786043. doi: 10.1155/omcl/7786043 (PMC12463520; doi:10.1155/omcl/7786043)

a

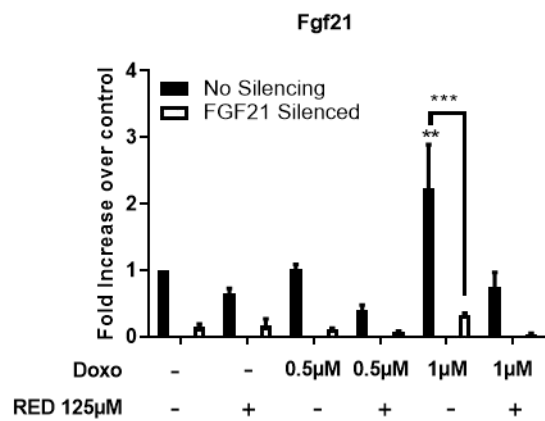

b

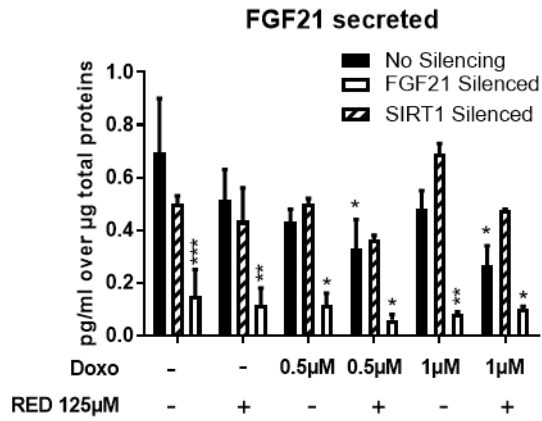

c

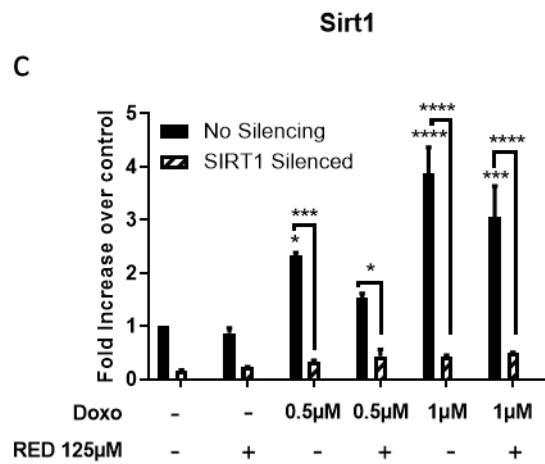

d

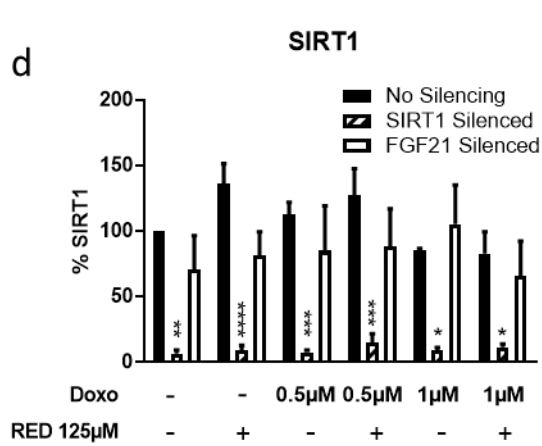

e

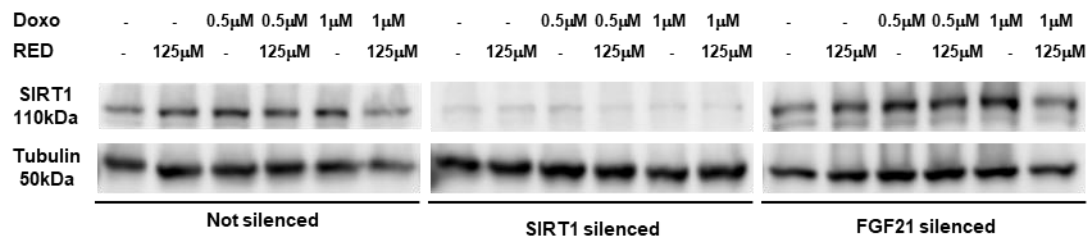

Supplement: Supporting Information 2 — Figure S1. Effect of Fgf21 and Sirt1 silencing on transcript and protein levels. qPCR analysis of the effects of Fgf21 and Sirt1 silencing on (a) Fgf21 and (c) Sirt1 transcript levels, respectively. Data are expressed as mean ± SEM of three technical replicates per condition from two independent biological experiments and were analyzed using two-way ANOVA followed by Tukey's multiple comparisons test. (b) ELISA assay showing the effect of Fgf21 and Sirt1 silencing on FGF21 secretion. Data are presented as mean ± SEM of two technical replicates per condition from two independent biological experiments and were analyzed using two-way ANOVA followed by Tukey's multiple comparisons test. (d, e) Western blot analysis of the effects of Fgf21 and Sirt1 silencing on SIRT1 in HL-1 cells treated with 0.5 or 1 µM Doxo with or without 125 µM RED. Data in Subpart (d) are presented as mean ± SEM of three independent biological experiments and were analyzed using two-way ANOVA followed by Sidak's multiple comparisons test. Subpart (e) shows a representative blot from one of the three independent biological replicates. ⁣∗p < 0.05, ⁣∗∗p < 0.01, ⁣∗∗∗p < 0.001, and ⁣∗∗∗∗p < 0.0001 indicate significant differences versus 0 µM Doxo and between indicated pairs. [file 7786043.f2.pdf]
